# Depletion of the LINC complex disrupts cytoskeleton dynamics and meiotic resumption in mouse oocytes

**DOI:** 10.1038/srep20408

**Published:** 2016-02-04

**Authors:** Yibo Luo, In-Won Lee, Yu-Jin Jo, Suk Namgoong, Nam-Hyung Kim

**Affiliations:** 1 Department of Animal Science, Chungbuk National University, Cheongju, Korea

## Abstract

The SUN (Sad-1/UNC-84) and KASH (Klarsicht/ANC-1/Syne/homology) proteins constitute the linker of nucleoskeleton and cytoskeleton (LINC) complex on the nuclear envelope. To date, the SUN1/KASH5 complex is known to function as meiotic-specific factors. In this study, gene-silencing methods were used to explore the roles of SUN1 and KASH5 in mouse oocytes after prophase. SUN1 was detected throughout the nucleus; however, KASH5 was dispersed through the cell. After germinal vesicle breakdown (GVBD), SUN1 and KASH5 migrated during spindle formation and localized to the spindle poles at the MII stage. Most oocytes were arrested at the germinal vesicle (GV) stage after depletion of either SUN1 or KASH5. The DNA damage response was triggered in SUN1-depleted oocytes and thus gave rise to the G2/M checkpoint protein, p-CHK1. Oocytes that underwent GVBD had relatively small and abnormal spindles and lower levels of cytoplasm F-actin mesh. Immunofluorescence results also indicated the dislocation of pericentrin and P150^Glued^ after SUN1 or KASH5 depletion. Furthermore, KASH5 localized exclusively near the oocyte cortex after SUN1 depletion, but SUN1 localization was unaffected in KASH5-depleted oocytes. Taken together, the results suggest that SUN1 and KASH5 are essential factors in the regulation of meiotic resumption and spindle formation.

The nuclear envelope partitions cytoplasmic and nuclear materials, which dissemble and reconstruct periodically during the cell cycle. However, channels or bridges are needed to ensure communication among the cytoskeleton, matrix, and molecules in the cytoplasm and nucleoplasm. The nuclear pore complex on the nuclear membrane functions as the channel for the passage of small proteins and nucleotides^1^, while the LINC complex, which is composed of the SUN (Sad-1/UNC-84) and KASH (Klarsicht/ANC-1/Syne/homology) domain proteins, acts as the bridge connecting the chromatin and cytoskeleton^2^. Evidence suggests that the LINC complex is also crucial for the movement and positioning of the nucleus both in animals and plants^3^,^4^. Of the members in the LINC complex, SUN domain proteins are always localized on the inner nuclear membrane (INM)^5^,^6^ and they tether chromosomes to the nuclear membrane through interactions with the telomeres^7^. KASH domain proteins, on the other hand, are localized on the outer nuclear membrane (ONM), and they anchor the dynein-dynactin complex and nuclear membrane^8^,^9^ Therefore, the LINC complex specifically forms the linkage between chromosomes and microtubules. SUN proteins in the LINC complex are evolutionarily conserved; in mammals, SUN1 and SUN2 are always found to be transmembrane proteins that interact with A-/B- type lamina inside the nucleus^10^. However, several KASH domain-containing proteins have been identified in different species, with different N-terminal sequences for each species.

As the membrane structure that links the nucleoplasm and the cytoplasm, the LINC complex is essential for the development of most tissues. For example, SUN/Nesprin4 double mutant mice have hearing deficiencies because the position of the nucleus in cells of the outer ear is severely affected^11^. Patients with the SUN1 mutation have muscular dystrophies because the low level of SUN1 reduces the connection of SUN1 to Lamin A/C and Emerin^12^,^13^. The LINC complex also regulates retinal development by mediating nuclear migration^14^. Therefore, it is evident that these defects resulting from mutations in the LINC complex are related to abnormalities in nuclear positioning and the nuclear skeleton, which also suggests that the LINC complex might have regulatory roles in the actin or microtubule cytoskeleton. Moreover, the KASH protein Emerin links the centrosome to the outer membrane of the nucleus^13^. Studies in fission yeast have demonstrated that telomere localization requires SUN proteins, which are gathered around the microtubule organizing center (MTOC) by microtubule associated motors^15^. Based on the results of these studies, it is likely that the LINC complex is closely related to cytoskeleton function.

During mitosis, SUN1 is phosphorylated by cyclin-dependent kinase 1 (CDK1) at serines 48 and 333 and by Polo-like kinase (PLK1) at serine 138. These phosphorylation events cause SUN1 to loosen its connection with lamin^16^, a key process that is required for nuclear envelope break down (NEBD). SUN proteins are also involved in the DNA damage response; results from studies investigating fibroblasts from SUN1/SUN2 double mutant mice showed severe DNA damage, apoptosis, and proliferated arrest in S phase^17^. SUN1/2 proteins are involved in the mobility of chromatin surrounding double-strand breaks in the DNA, further indicating the roles of the LINC complex in DNA damage repair^18^.

Recently, several reports on the role of the LINC complex in mammalian male meiosis have emerged^8^,^9^. In mice, SUN1 and SUN2 are the only SUN domain proteins that are expressed during meiosis, and they are co-localized with the meiosis-specific protein KASH5, also known as CCDC155^8^. At the onset of meiosis, SUN proteins localize on telomeres and regulate chromatin combination and synaptonemal complex formation. SUN1 or KASH5 knockout mice are infertile because pairing between homologous chromosomes does not occur, and meiosis is arrested at the leptotene/zygotene stage of meiotic prophase I in spermatocytes^7^,^9^.

During mammalian female germ cell development, oocytes are arrested at prophase I after birth, and germinal vesicle breakdown (GVBD) signals the resumption of meiosis, followed by chromosomal alignment and spindle formation^19^. During this process, a series of phosphorylation and de-phosphorylation events occur in the germinal vesicle, and eventually, the Lamin protein on the nuclear membrane is phosphorylated to mediate GVBD^20^,^21^. Furthermore, given the LINC complex is located on the nuclear membrane and provides a bridge between the nuclear envelope and microtubule cytoskeleton, the complex could play roles in GVBD or meiotic spindle formation in oocyte maturation. However, such roles of the LINC complex in oocyte maturation have not been investigated. Here, we report the localization of SUN1 and KASH5 in mouse oocytes after prophase and determine the LINC complex’s role in meiotic resumption and spindle formation.

## Results

### Localization of SUN1 and KASH5 during meiotic maturation in mouse oocytes

**Figure 1:**
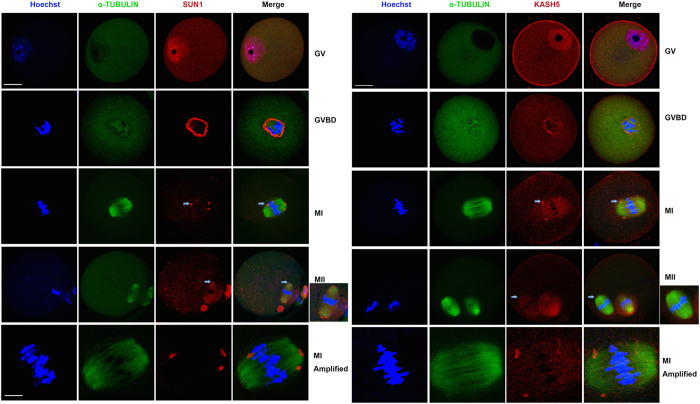
Subcellular localization of SUN1 and KASH5 in mouse oocytes. (**A**) Localization of SUN1 during mouse oocyte meiotic maturation. From top to bottom, the rows are indicated as: GV, GVBD and MI, MII stage and image of MI spindle. Localization of SUN1 as speckles near the MI spindle and spindle poles is indicated as an arrow. DNA: Blue; α-Tubulin: Green; SUN1: Red. (**B**) Localization of KASH5 during mouse oocyte meiotic maturation. Arrows indicate the KASH5 localization as speckles near the spindle at the MI stage and the spindle pole localization at the MII stage. DNA: Blue; α-Tubulin: Green; KASH5: Red. The scale bar is 20 μM for the oocytes images and 5 μM for the MI spindle at the bottom row.

First, we examined the subcellular localization of SUN1 and KASH5 during mouse oocyte maturation. Oocytes were sampled at 0, 2, 8, or 12 h after starting *in vitro* cultures, which corresponded to the germinal vesicle (GV), GVBD, metaphase I (MI), or metaphase II (MII) stage, respectively. Using confocal immunofluorescence microscopy, we detected subcellular localization of SUN1 and KASH5 (Fig. 1). At the GV stage, SUN1 predominantly localized throughout the nucleus. SUN1 signals were also found in the cytoplasm, although its signal intensity was far weaker than those in the nucleus. After GVBD, intense signals appeared around the chromosome and colocalized with α-tubulin. At the MI stage, the SUN1 signal was detected as small dots around the meiotic spindle; after first polar body extrusion and the MII stage, SUN1 mainly localized at the spindle poles (Fig. 1A). KASH5 appeared to mainly have the same localization patterns as SUN1. At the GV stage, KASH5 also localized to the nucleus. After GVBD, KASH5 localized around the α-tubulin, and at the MI stage, dot signals could also be found around the meiotic spindle. At the MII stage, KASH5 localized to the poles of the spindle. However, unlike SUN1, KASH5 localized to the oocyte membrane throughout all meiotic stages (Fig. 1B).

To confirm specificity of the antibodies against SUN1 and KASH5 and observed localizations, we also stained SUN1 and KASH5 in mouse embryonic fibroblasts (MEF) using the same antibodies. Confocal microscopy results indicated that in somatic cells, SUN1 localized to the inner nuclear membrane, and KASH5 localized to the outside of the nucleus. Collectively, these observations are consistent with the findings in mitosis (Supplementary Fig. S1). Hence, the findings indicate that SUN1 and KASH5 are differentially localized in germ (oocytes) and somatic cells (MEFs).

### Depletion of SUN1 or KASH5 resulted in the abnormal development of mouse oocytes

**Figure 2:**
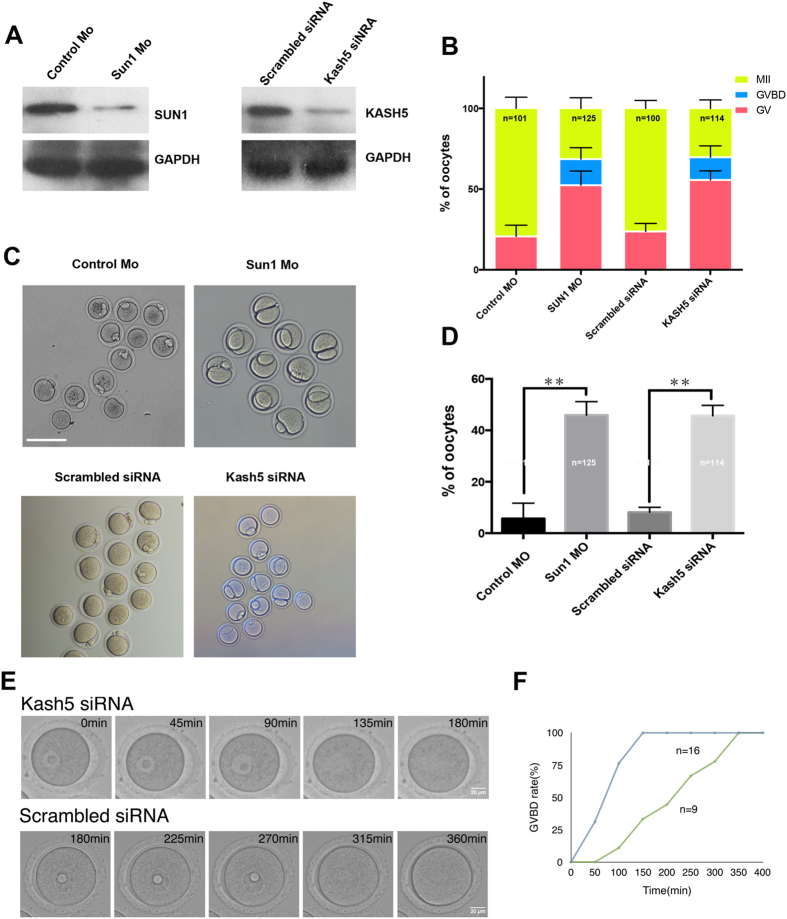
Depletion of SUN1 or KASH5 affects mouse oocyte maturation. (**A**) Western blots analysis of the knockdown of SUN1 and KASH5 proteins in mouse oocytes by SUN1 Morpholino and KASH5 siRNA microinjection. Microinjected oocytes were arrested at GV stages for 24 h in the presence of milrinone, and 150 oocytes for each treatment were collected and analyzed. (**B**) Oocyte maturation was affected by SUN1 or KASH5 depletion, with most of the oocytes arresting at the GV stage (p < 0.001). (**C**,**D**) SUN1 (45.9%) or KASH5 (45.6%) depleted oocytes with first polar body extrusion underwent asymmetric division or produced a larger polar body, while 6% and 5% (p < 0.001) of the control-injected oocytes respectively displayed any abnormalities. Bar = 100 μM. (**E,F**) Delayed GVBD in KASH5 knockdown oocytes. KASH5 or scrambled siRNA was injected, and GVBD timing was measured by time-lapse microscopy. Bar  = 20 μM. Time course of GVBD in KASH5 knockdown (n = 13) or Control (n = 9) oocytes was observed, and GVBD rates were measured for each time course analysis. Only oocytes that underwent GVBD in the time-lapse microscopy were analyzed.

We used RNAi or morpholino antisense oligonucleotide-mediated gene expression knockdown to further characterize the functional roles of SUN1 and KASH5 in oocyte maturation. For this, 150 oocytes (microinjected to deplete either SUN1 or KASH5) were arrested in the GV stage for 24 h by milrinone and collected afterwards for western blot analysis to assess SUN1 and KASH5 protein levels. Microinjections of SUN1 morpholino and KASH5 siRNA decreased protein levels of SUN1 and KASH5, respectively (Fig. 2A). After GV arrest in 2.5 μM milrinone for 24 h, followed by 12 h culture in M2 medium, only 31 ± 6% of oocytes microinjected with the SUN1 morpholino and 79 ± 7% of the control oocytes (injected with control morpholino) extruded the first polar body (p < 0.01). However, 52 ± 9% of SUN1-depleted oocytes were arrested at the GV stage, and in the SUN1 morpholino injected group, 21 ± 7% (p < 0.01) were arrested at the GV stage. Moreover, in 16 ± 7% oocytes, maturation stopped after GVBD without first polar body extrusion in the SUN1 depletion group.

Similar events occurred in KASH5-depleted oocytes. siRNA targeting KASH5 mRNA was injected into the mouse oocytes at the GV stage. Approximately 55 ± 5% of oocytes injected with KASH siRNA showed arrest at the GV stage, and only 30 ± 5% extruded the first polar body. In the control group (scrambled siRNA injected group), 23 ± 5% and 76 ± 5% of the oocytes showed arrest at GV stage and extruded the first polar body, respectively (p < 0.01). Furthermore, 14  ± 7% of the siRNA injected oocytes, but none of the control oocytes, underwent GVBD without first polar body extrusion (Fig. 2B).

In addition to these abnormalities, 46 ± 3% and 46 ± 2% oocytes that reached the MII stage in the SUN1-depleted and KASH5-depleted groups, respectively, had large polar bodies with symmetric division. In contrast, only 6 ± 3% of oocytes from the control morpholino group and 4 ± 3% of oocytes from the scrambled siRNA group had large polar bodies (Fig. 2C,D).

While half of the oocytes in the KASH5-depleted group showed arrest at the GVBD stage (Fig. 2B), the other half developed beyond GVBD. Using time-lapse microscopy, we observed the timing of GVBD in control and KASH5 knockdown oocytes. Even though some of the KASH5-depleted oocytes matured beyond GVBD, the GVBD timing was slower than those in the control oocyte group (Fig. 2E,F). For example, at 150 min after meiosis resumption, GVBD occurred in 100% of control oocytes, but only 33% of oocytes went beyond GVBD in the KASH5-depleted group. These results indicate that ablation of KASH5 levels in oocytes impair GVBD.

### Depletion of SUN1 triggered the DNA damage response

**Figure 3:**
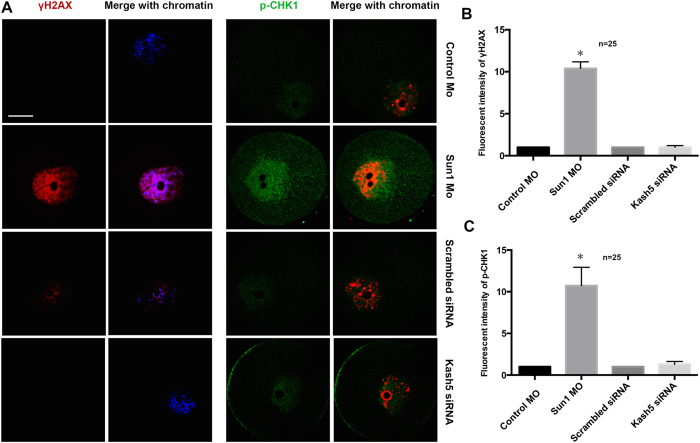
SUN1 but not KASH5 depletion triggers the DNA damage response. (**A**) Localization of γH2AX and p-CHK1 in the nuclei of GV stage oocytes with either SUN1 or KASH5 knocked down or respective controls. Red: γH2AX; Blue: DNA; Green: p-CHK1. (**B**) Quantification of γH2AX levels in nuclei determined by relative fluorescence intensity in SUN1 or KASH5 knockdown groups and respective controls. (**C**) Quantification of p-CHK1 levels in nuclei determined by relative fluorescence in SUN1 or KASH5 knockdown groups and respective controls.

The depletion of SUN1 or KASH5 resulted in the aberrant development of mouse oocytes, and most of the oocytes were arrested at the GV stage. We next decided to investigate the effect of SUN1 or KASH5 depletion on the cell cycle checkpoint. Therefore, we stained γH2AX, marker for double strand DNA break^22^ and its downstream G2/M checkpoint protein, p-CHK1^23^, in SUN1 or KASH5-depleted mouse oocytes. γH2AX and p-CHK1 signals increased in SUN1-depleted oocytes, suggesting that SUN1 was associated with the DNA damage response (Fig. 3A, left). However, depletion of KASH5 in mouse oocytes did not increase the γH2AX and p-CHK1 signals (Fig. 3A, right), and the fluorescence intensity was not significantly different from that of control oocytes (Fig. 3B,C). Thus, SUN1, but not KASH5, functions in the DNA damage response in mouse oocytes.

Furthermore, we counted the oocytes that showed positive γH2AX and p-CHK1 signals after SUN1 depletion. The statistical results demonstrated that 14 ± 4% of control oocytes and 57 ± 5% of SUN1-depleted oocytes presented a positive γH2AX signal. Furthermore, 15 ± 2% in the control group and 59 ± 2% in the SUN1-depleted group displayed positive p-CHK1 signals. The percent of oocytes with positive γH2AX and p-CHK1 signals corresponded to the number of GV-arrested oocytes (Supplementary Fig. S3A).

### Depletion of SUN1 or KASH5 in mouse oocytes disordered spindle formation and cytoplasmic actin density

**Figure 4:**
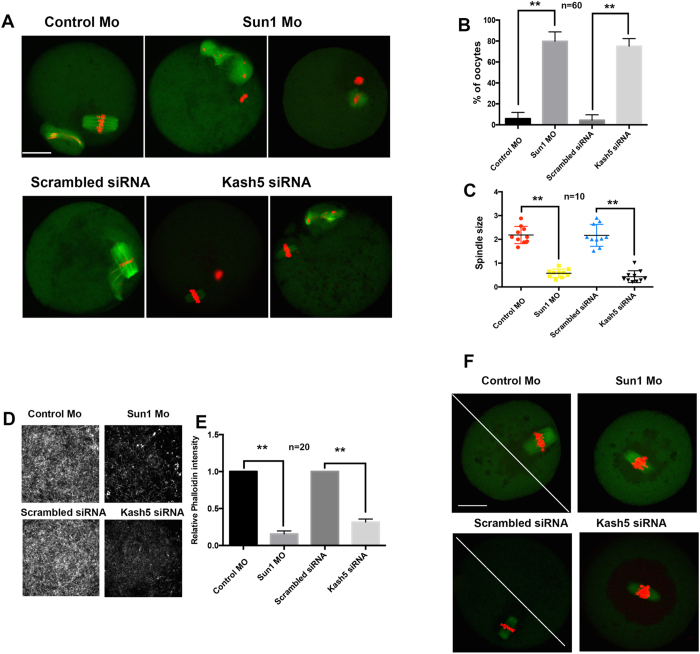
SUN1 or KASH5 depletion leads to the abnormal spindle formation and altered F-actin intensity. (**A**) Abnormal spindle formation after SUN1 (upper row) or KASH5 (lower row) depletion. Green: α-tubulin; Red: chromatin. Bar = 20 μM. (**B**) Ratio of polar body extruded oocytes possessing abnormal spindles after SUN1 or KASH5 knockdown. (**C**) Spindle size quantifications in SUN1 or KASH5-depleted oocytes. Length of spindle (μm) was measured. (**D,E**) Cytoplasmic F-actin density decreased in SUN1 or KASH5-depleted oocytes. (**F**) Spindle location after 7 h of meiotic resumptions in SUN1 or KASH5-depleted oocyte. Green: α-tubulin; Red: chromatin.

During the MI and MII stages in mouse oocytes, SUN1/KASH5 localized around the spindle or to the spindle poles, which implied that the LINC complex affected spindle formation or function. In this study, very few oocytes extruded the first polar body, and nearly half of the MII oocytes underwent aberrant division patterns after SUN1/KASH5 depletion. In addition, we checked the effect of SUN1 or KASH5 depletion on meiotic spindle formation. As shown in Fig. 4, 80 ± 6% or 75 ± 4% of SUN1 or KASH5-depleted oocytes formed abnormal spindles; only 6 ± 3% and 4 ± 3% of control groups showed abnormality in the spindle (Fig. 4A,B). Furthermore, the spindle sizes were relatively smaller than the sizes of oocytes from the control group (0.6 μM vs. 2.2 μM for SUN1 and control groups and 0.4 μM vs. 2.2 μM for KASH5 and control groups; p < 0.01; Fig. 4C).

Because we observed frequent failures of asymmetric division in SUN1 or KASH5-depleted groups, we examined the effect of SUN1 or KASH5 depletions on cytoplasmic actin mesh density, which has been known to be involved in spindle migration and asymmetric division^24^,^25^. Results clearly indicated that in SUN1 or KASH5-depleted oocytes, F-actin intensity was significantly lower than in control morpholino or scrambled siRNA injected oocytes (15% of the control intensity for SUN1 depletion and 20% for KASH5 depletion; Fig. 4D).

Oocyte asymmetric division requires the spindle migration near the cortex at MI, which needs the normal traction by actin. As F-actin mesh level in cytoplasm was decreased in SUN1 or KASH5 depleted oocytes, the spindle migration would also be affected. Therefore, we labeled spindles with α-tubulin-FITC to determine the spindle position at 7 h in control and SUN1/KASH5 knockdown groups. The results demonstrated that in the control oocytes, the majority of the spindles migrated near the cortex and at least moved out of the oocyte center. However, in 67 ± 5% of SUN1 or 64 ± 10% KASH5-depleted oocytes, the spindles remained around the center of oocytes, compared to the data from the control groups (13 ± 3% and 16 ± 6%, respectively; Fig. 4F and Supplementary Fig. S3B).

### Depletion of SUN1 or KASH5 prevented spindle pole localization of Pericentrin and P150 in mouse oocytes

**Figure 5:**
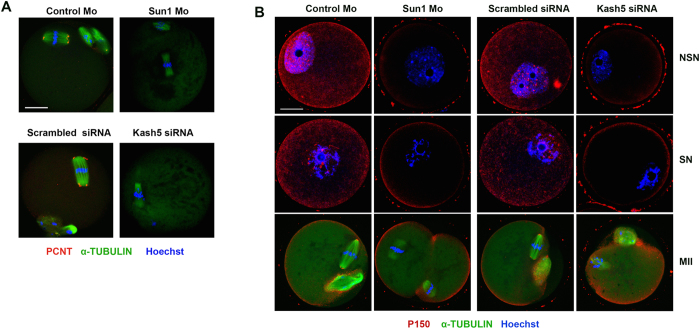
The localization patterns of PCNT and P150 are altered after SUN1 or KASH5 depletion. (**A**) PCNT is not recruited to the spindle poles after SUN1 or KASH5 depletion. (**B**) P150 localized exclusively to the oocyte cortex in SUN1 or KASH5 depleted oocytes. In control oocytes, P150 localized as dots at the GV stage and to spindle poles at the MII stage. Bar = 20 μM.

To understand the mechanism of meiotic spindle abnormality caused by SUN1/KASH5 depletion, we determined the localization of Pericentrin (PCNT)^26^, in SUN1/KASH5-depleted mouse oocytes. PCNT is the key MTOC-associated protein in oocytes and is essential for normal meiotic spindle formation^27^. At the MI stage, PCNT localized as dots to the spindles (Supplementary Fig. S2A). At the MII stage, PCNT accumulated on the spindle poles, similar to the localization pattern of SUN1/KASH5. However, after SUN1 or KASH5 depletion, PCNT was not detected at the spindle poles, which correlated with the aberrant spindle formation after SUN1 or KASH5 depletion (Fig. 5A).

Besides PCNT, dynein/dynactin is also associated with the normal spindle formation during the cell cycle, and usually PCNT recruitment is dynein-dependent^28^. Furthermore, KASH5 has been reported to interact with dynactin^8^,^9^, therefore providing a connection between the microtubule cytoskeleton and LINC complex. Therefore, we examined the changes in dynactin after SUN1 or KASH5-depletion. Dynein/dynactin is a complex of several factors; therefore, we chose to examine P150^Glued^ 29, which is the largest factor involved in the dynein/dynactin complex.

In normal mouse oocytes, at the non-surrounding nucleolus (NSN) stage of the GV stage, P150 ^Glued^ localized to the nucleus as small dots and around the cortex, and at the surrounding nucleolus (SN) stage of GV stage, P150 formed large dots in the nucleus and close to the cortex. After SUN1 or KASH5-depletion, however, nuclear localization of P150^Glued^ was not detected. At the MII stage, P150^Glued^ was also localized to the spindle poles; however, in SUN1 or KASH5-depleted mouse oocytes, there was no spindle pole signal, and most of the P150^Glued^ signal was detected in the cytoplasm, suggesting abnormal localization of dynein/dynactin complex after SUN1/KASH5 depletion (Fig. 5B).

### Nuclear localization of KASH5 is dependent of the presence of SUN1 in mouse oocytes

**Figure 6:**
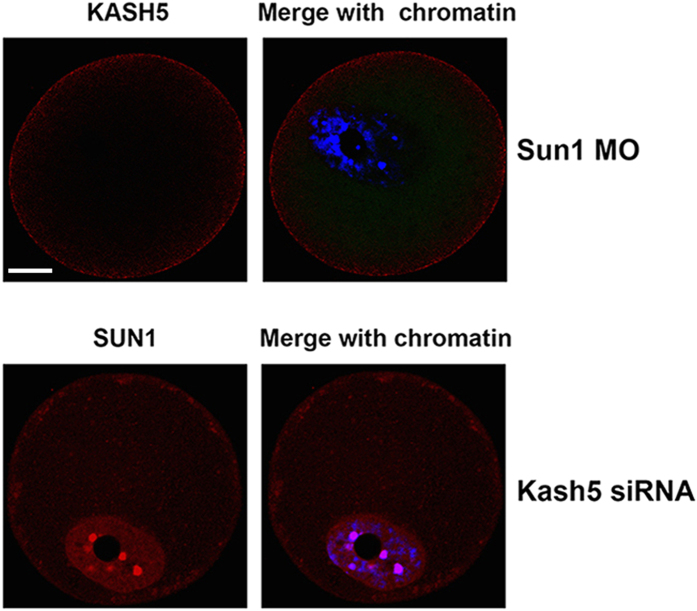
Nuclear localization of KASH5 is dependent on SUN1. Upper panels show the exclusive localization of KASH5 to the cortex after SUN1 depletion. Lower panels show that nuclear localization of SUN1 is unaffected by KASH5 depletion. Bar = 20 μM.

Since SUN1 and KASH5 form a complex, it is expected that one member would be affected if the other member was depleted. We microinjected the SUN1 morpholino into mouse oocytes at the GV stage to inhibit the translation of SUN1 protein and then examined KASH5 localization. We found that in SUN1-depleted oocytes KASH5 was not detected in the nucleus. The KASH5 signal only appeared weakly at the cortex region, which suggested that nuclear localization of KASH5 is dependent on the presence of SUN1 in mouse oocytes (Fig. 6). Surprisingly, after KASH5 was depleted through siRNA microinjection, SUN1 localization in the nucleus was unaffected in mouse oocytes at the GV stage. Fluorescence intensity was not significantly affected when compared to the intensity in control oocytes (Fig. 6).

## Discussion

The nuclear envelope separates the cytoplasm and the nucleoplasm and serves as a bridge between the cytoskeleton and nuclear skeleton. In somatic cells, NEBD and reassembling are the main procedures involved in cell division and duplication. In germ cells and zygotes, NEBD is also essential for the meiotic resumption and pronuclear migration^30^. Being one of most important protein complexes localized on the nuclear envelope, elucidating the impact of the LINC complex on cellular function is essential.

During mitosis, SUN1 localizes to the inner nuclear membrane while KASH5 localizes to the outer nuclear membrane, as indicated in previous reports^7^,^9^ and by our studies on MEFs. However, in mouse oocytes, SUN1 and KASH5 overall co-localized in the nucleus, and the KASH5 signal was also detected near the oocyte cortex. This specific localization suggests specific roles of the LINC complex in mouse oocytes. In this study, we also stained SUN1 and KASH5 in mouse pronuclei and early stage embryos. Strikingly, the results suggested that SUN1 was localized to the whole nucleus while KASH5 was localized to the outer membrane of nucleus, which was a different expression pattern from that in oocytes. The underlying mechanism of this change in KASH5 localization needs to be further investigated.

After GVBD, the LINC complex associated with the meiotic spindle. At MI, SUN1 and KASH5 appeared as dots around the forming spindle, and at MII stage, they both localized dominantly to the spindle poles. This is a novel finding, which suggests that the LINC complex might influence spindle function in mouse oocytes. The results from our loss-of-function experiments verified this deduction. In SUN1 or KASH5-depleted oocytes, the spindles were relatively small, and the percentage of first polar body extrusion by the depleted oocytes was significantly reduced. As previously indicated, the LINC complex bridges the cytoskeleton with the nuclear-skeleton. In the outer nuclear membrane, KASH5 was associated with the cytoskeleton via dynein-dynactin^8^,^9^, which allowed the centrosome factors to move along the microtubulin. In spermatocytes, dynein modulates chromosome movement and pairing and, consequently, affects meiotic progress through interactions with the LINC complex. In contrast to spermatocytes, where KASH5 is absent in MI^8^, we observed SUN1/KASH5 localization in the meiotic spindle of oocyte MI, and it may reflect one of the differences in male and female gamete formation. Our results further demonstrated that, after depletion of SUN1 or KASH5, the nuclear localization of P150^Glued^ was altered at the GV stage, however spindle pole localization of P150 ^Glued^ was eliminated at the MII stage. It is known that dynein plays a number of roles during normal spindle formation. For example, P150^Glued^ localizes to the mother centriole at the G1/S stage, and its depletion down-regulates the expression of cyclin B1^31^. It is also reported that spindle assemble checkpoint proteins that move from the kinetochores towards the spindle poles are dynein-dependent^32^,^33^.

Our results that SUN1 or KASH5 knockdown caused spindle abnormality, and depletion of PCNT or P150^Glued^ localization implied that the LINC complex plays roles in meiotic spindle formations possibly via the dynein/dynactin complex and acentriolar microtubule-organizing centers (MTOCs), which have been known to be involved in the formations of meiotic spindles^34^. A recent report suggested that dynein is involved in stretching MTOCs along the nuclear membranes^28^. SUN1/KASH5 could link dynein/dynactin with the nuclear membrane and facilitate GVBD and the meiotic spindle. The exact molecular mechanism of SUN1/KASH5 in spindle formations and GVBD warrant further investigations.

Previously, SUN1 has been associated with the DNA damage response during mitosis^17^,^18^. Particularly, SUN1 attaches the telomeres of chromosomes to the nuclear envelope, and depletion of SUN proteins triggers the DNA damage response. In the present study, we found that in mouse oocytes, depletion of SUN1 up-regulates the expression of γH2X and the G2/M checkpoint protein, p-CHK1, and subsequently causes oocyte arrest at the meiotic GV stage. It was previously reported that high levels of SUN proteins leads to a hyperactive DNA damage response that is a major cause of laminapathies^17^. Thus, it is likely that during normal cell function, the amount of SUN proteins must be tightly regulated. However, in contrast to SUN1, depletion of KASH5 could not trigger the DNA damage response, as indicated by the failure of KASH5 depletion to affect γH2X and p-CHK1 levels. Nevertheless, KASH5 depletion caused oocyte arrest during meiotic maturation at the GV stage as well. We suggest that this effect was due to the severe impact of KASH5 depletion on the cytoskeleton, since the spindle was relatively small and F-actin intensity was significantly reduced. In fact, the destruction of microtubulin and actin could inhibit cellular growth. In kangaroo rat kidney cell lines, destroying microtubulin delays the entry of late G2 cells to prometaphase^35^. At late G2, microtubulin accumulates near the nuclear envelope, suggesting that microtubule may interact with nuclear envelope proteins.

KASH5 stabilization in mouse oocytes requires the presence of SUN1; however, depletion of KASH5 did not alter the expression pattern of SUN1. Haque *et al*. found that during mitosis SUN1 localizes correctly even when it lacks its KASH partner, Emerin^12^. These results imply the possibility that SUN1 would interact with another partner in oocytes. SUN1/SUN2 have been known to interact with other KASH domain proteins, including Syne/Nesprin-1/2 in neurons^36^. Besides the KASH domain, Nesprin-1 and Nesprin-2 have F-actin binding domains, and therefore these proteins may provide a link between the nuclear envelope and actin cytoskeleton^37^. A link between SUN1 with actin-binding Nesprin-1 and Nesprin-2 may explain the ablations of cytoplasmic actin by SUN1 or KASH5 depletions, although involvement of Nesprins in oocyte maturation and their modulation of the actin cytoskeleton in oocyte maturation need to be investigated further.

In conclusion, we report the meiosis-specific localization of SUN1 and KASH5 in mouse oocytes; we found that depletion of both SUN1 and KASH5 caused defects in various stages of oocyte maturation, including failure of GVBD, abnormal spindle formation, and low levels of cytoplasmic F-actin. Our study suggests novel roles of the LINC complex during oocyte maturation. Particularly, the meiosis specific LINC complex may play crucial roles in the final step of female gamete formation as well as spermatogenesis.

## Methods

### Ethics statement

The mouse strain, ICR, was used in all experiments. Animal care and handling and experiments were conducted in accordance with policies issued by the ethical committee of the Department of Animal Science, Chungbuk National University. In addition, all experimental protocols were approved by the ethical committee of the Department of Animal Science, Chungbuk National University.

### Oocytes collection

To collect the oocytes for *in vitro* maturation (IVM), ovaries were removed from 6- to 8 week-old mice and were chopped into pieces with a razor blade. The debris was dispersed with M2 medium (Sigma, St Louis, MO, USA). Oocytes that were spherical and of similar size were collected and then cultured in M2 medium. Oocytes used for microinjection were cultured in M2 medium supplemented with 2.5 μM milrinone to maintain them at the GV stage during microinjection. After microinjection, oocytes were washed thoroughly and cultured in M2 medium and covered with liquid paraffin oil at 37 °C in the presence of 5% CO_2_ until they reached the GV (0 h), GVBD (2 h), MI (8 h), or MII (12–14 h) stage.

### Microinjection of SUN1 morpholino oligomers and KASH5 siRNA

Microinjection was performed using an Eppendorf microinjector (Hamburg, Germany) and was completed within 1 h. The SUN1-targeting morpholino and its control were purchased from Gene Tools (Philomath, OR, USA). The morpholino was diluted to 1 mM and microinjected into GV-stage oocytes. After microinjection, the oocytes were cultured for 24 h in M2 medium supplemented with 2.5 μM milrinone to maintain the oocytes at the GV stage and to ensure that the morpholino inhibited the translation of SUN1. As a control, GV-stage oocytes were injected with the control morpholino and cultured under the same conditions. KASH5 siRNA was purchased from Bioneer (Seoul, Korea). The oocytes microinjected with KASH5 siRNA were similarly handled as oocytes that were injected with the SUN1 morpholino. As the control, scrambled siRNA was injected in mouse oocytes, which were cultured under the same conditions.

### Antibodies and immunofluorescence

Antibodies detecting SUN1 and P150 were purchased from Abcam (Cambridge, UK). Antibodies detecting KASH5 and p-CHK1 were purchased from Santa Cruz Biotechnology (Santa Cruz, CA, USA). An antibody detecting γH2A.X was purchased from Cell Signaling (Boston, MA, USA). An antibody detecting PCNT was purchased from BD Bioscience (Franklin Lake, NJ, USA). Immunofluorescence staining was performed using standard protocols. Briefly, oocytes were fixed in 4% paraformaldehyde for 30 min and then treated with 0.5% Triton X-100 for 20 min. After blocking in 1% bovine serum albumin (BSA) for 1 h, oocytes were incubated with the primary antibody (1:50) at 4 °C overnight. After five washes in phosphate-buffered saline (PBS) containing 0.05% Tween 20 (washing buffer), oocytes were incubated with the secondary antibody conjugated with TRITC or FITC (diluted 1:200 in washing buffer, Life Technologies, Carlsbad, CA, USA) for 1 h at room temperature. Thereafter, the oocytes were washed four times in washing buffer and stained with a FITC-anti-α-tubulin antibody (diluted 1:1000 in PBS, Sigma) in order to detect the spindle. If F-actin intensity was measured, the oocytes were labeled with phalloidin-TRITC (Sigma, P1591, 5 μg/mL) for 2 h. After four washes in washing buffer, oocytes were incubated for 15 min with Hoechst 33342 dye (0.5 μg/mL) prepared in PBS. Finally, oocytes were mounted onto glass slides and examined using a laser scanning confocal microscope (Zeiss LSM 710 META, Oberkochen, Germany). The cytoplasmic F-actin mesh level was determined as previously described^38^,^39^. Briefly, oocytes in the MI stage were stained with phalloidin-TRITC, which labels F-actin. Oil immersion objective lens (63×) were used for observation. Fluorescence intensity in cytoplasmic regions devoid of the cortex was measured using ImageJ^40^.

### Western blotting

For Western blotting, mouse oocytes were collected in SDS sample buffer and heated for 5 min at 100 °C. Proteins were separated by SDS-PAGE and electrically transferred to polyvinylidene fluoride (PVDF) membranes. Membranes were blocked in TBST (Tris-Buffered Saline with Tween 20) containing 5% BSA for 2 h and then were incubated overnight at 4 °C with the primary antibody (1:500). After washing three times in TBST (each for 10 min), membranes were incubated for 1 h at 37 °C with a peroxidase-conjugated secondary antibody (1:2,000, Santa Cruz, CA, USA). Finally, membranes were processed using the SuperSignal West Femto Maximum Sensitivity Substrate (Thermo Scientific, Waltham, MA, USA).

### Time-lapse microscopy of oocyte maturation

Time-lapse imaging was performed in KASH5-depleted oocytes. siRNA against KASH5 or scrambled siRNA was microinjected into GV-stage oocytes. Images were captured at 300 s intervals for 6 h after resumption of meiosis using the Lumascope 620 (Etaluma Inc., Carlsbad, CA, USA) inverted microscope installed inside an incubator maintained at 37 °C and 5% CO_2_.

### Statistical analysis

All percentage data were subjected to arcsine transformation prior to statistical analysis and presented as the average  ± S.E.M. Data were analyzed by the t-test using the SPSS software package. p < 0.05 was considered statistically significant.

## Additional Information

**How to cite this article**: Luo, Y. *et al*. Depletion of the LINC complex disrupts cytoskeleton dynamics and meiotic resumption in mouse oocytes. *Sci. Rep.* **6**, 20408; doi: 10.1038/srep20408 (2016).

## Supplementary Material

Supplementary Information
