# Supplementary material for: Depletion of the LINC complex disrupts cytoskeleton dynamics and meiotic resumption in mouse oocytes
Source: Sci Rep. 2016 Feb 4;6:20408. doi: 10.1038/srep20408 (PMC4740751; doi:10.1038/srep20408)
Supplement: Supplementary Information [file srep20408-s1.pdf]

Supplementary figures

Depletion of LINC complex disrupts cytoskeleton dynamics and meiotic resumption in mouse oocytes

Yibo Luo, In-Won Lee, Yu-Jin Jo, Suk Namgoong and Nam-Hyung Kim*
Department of Animal Science, Chungbuk National University, Cheongju, Korea
*Correspondence: Nam-Hyung Kim.
Tel: +82-43-261-2546
Fax: +82-43-272-8853
E-mail: nhkim@chungbuk.ac.kr

Supplementary Figure S1

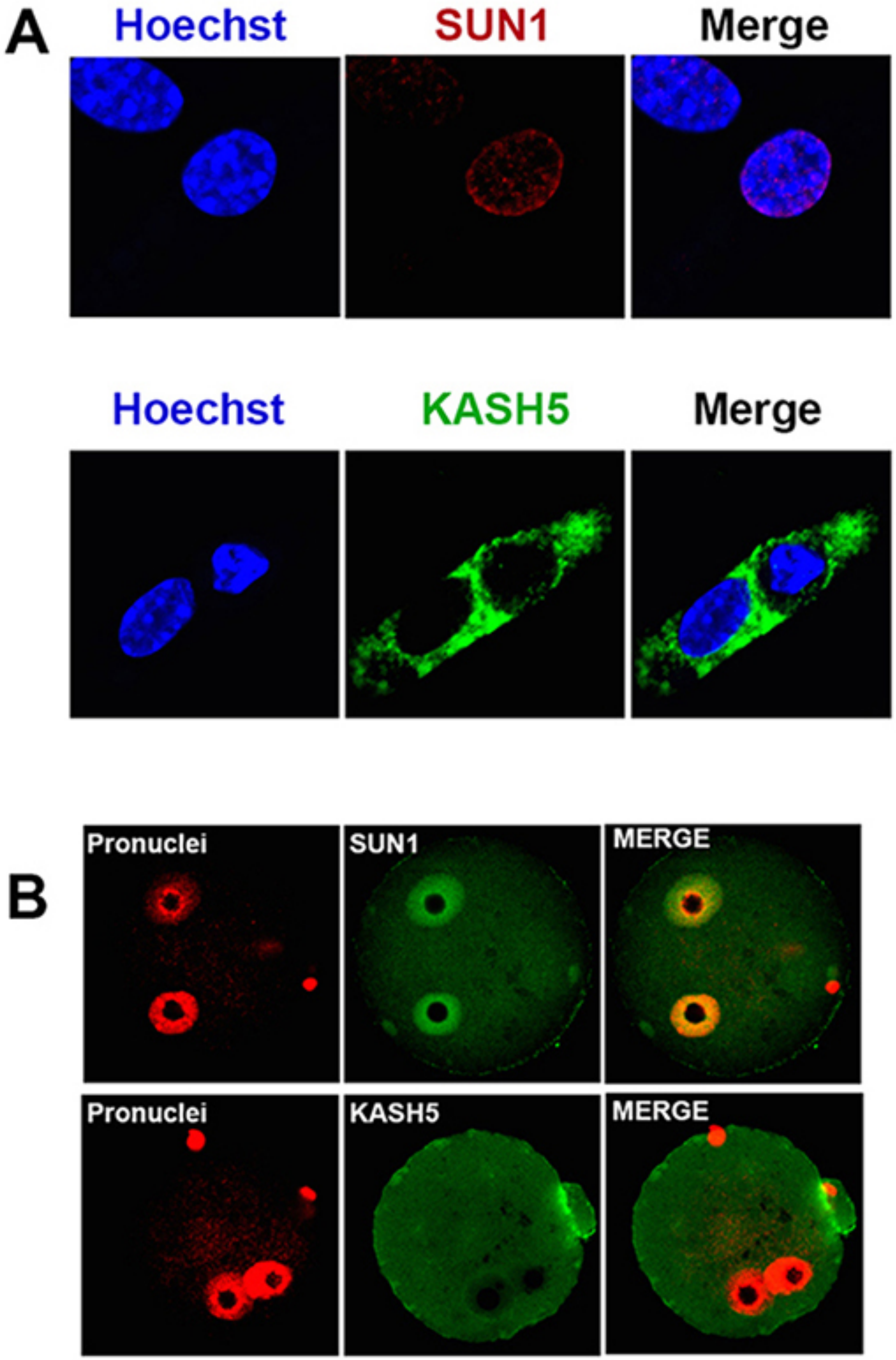

Figure S1. Localization of SUN1 and KASH5 in (A) mouse embryonic fibroblasts (MEF) and (B) zygotes at pronuclei stage. Bar=20µm.

Supplementary Figure S2

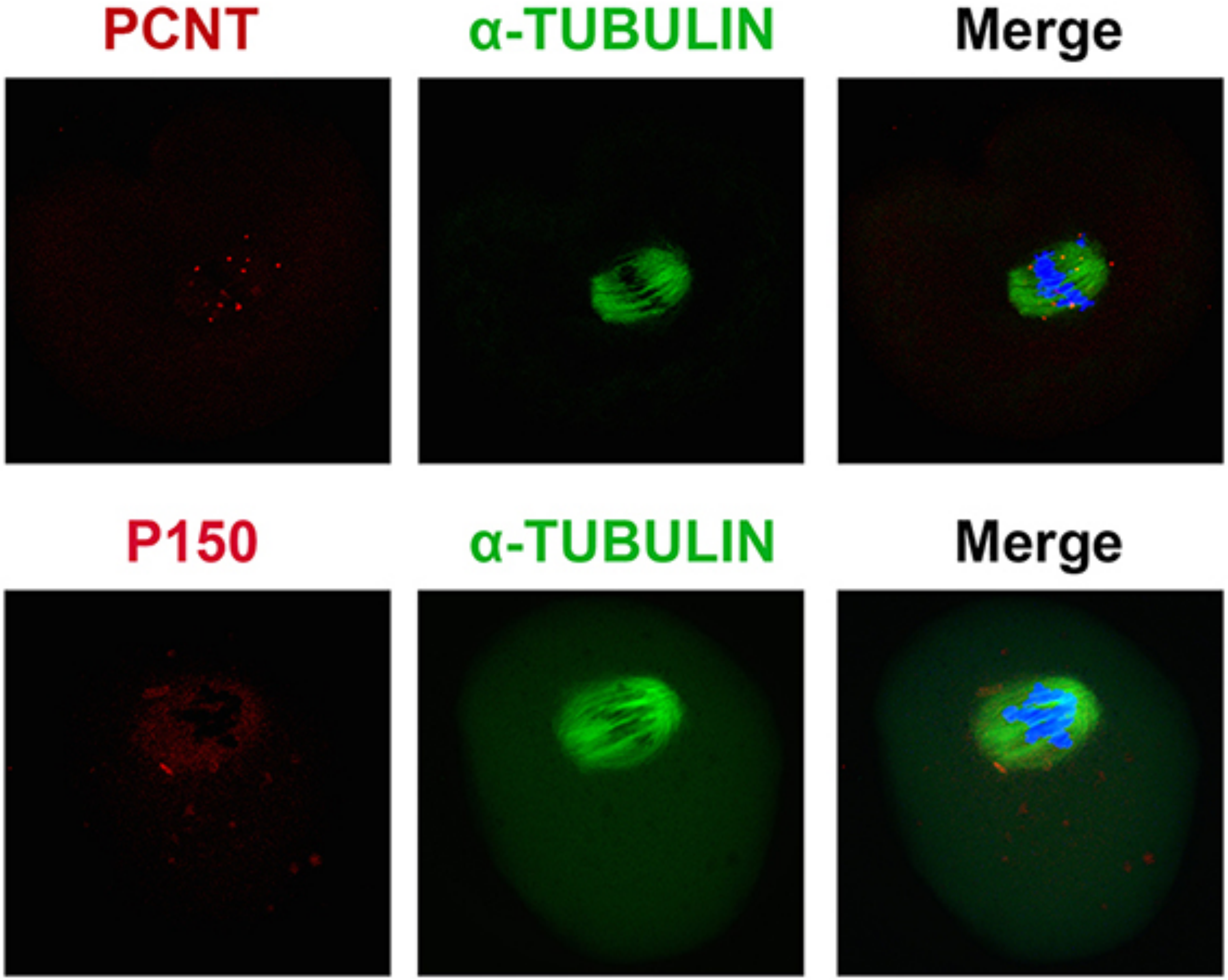

Figure S2. Localization of (A) PCNT and (B) P150 at MI stage. PCNT and P150 both show dot signals around the spindle at MI stage. Bar=20μm.

Supplementary Figure S3

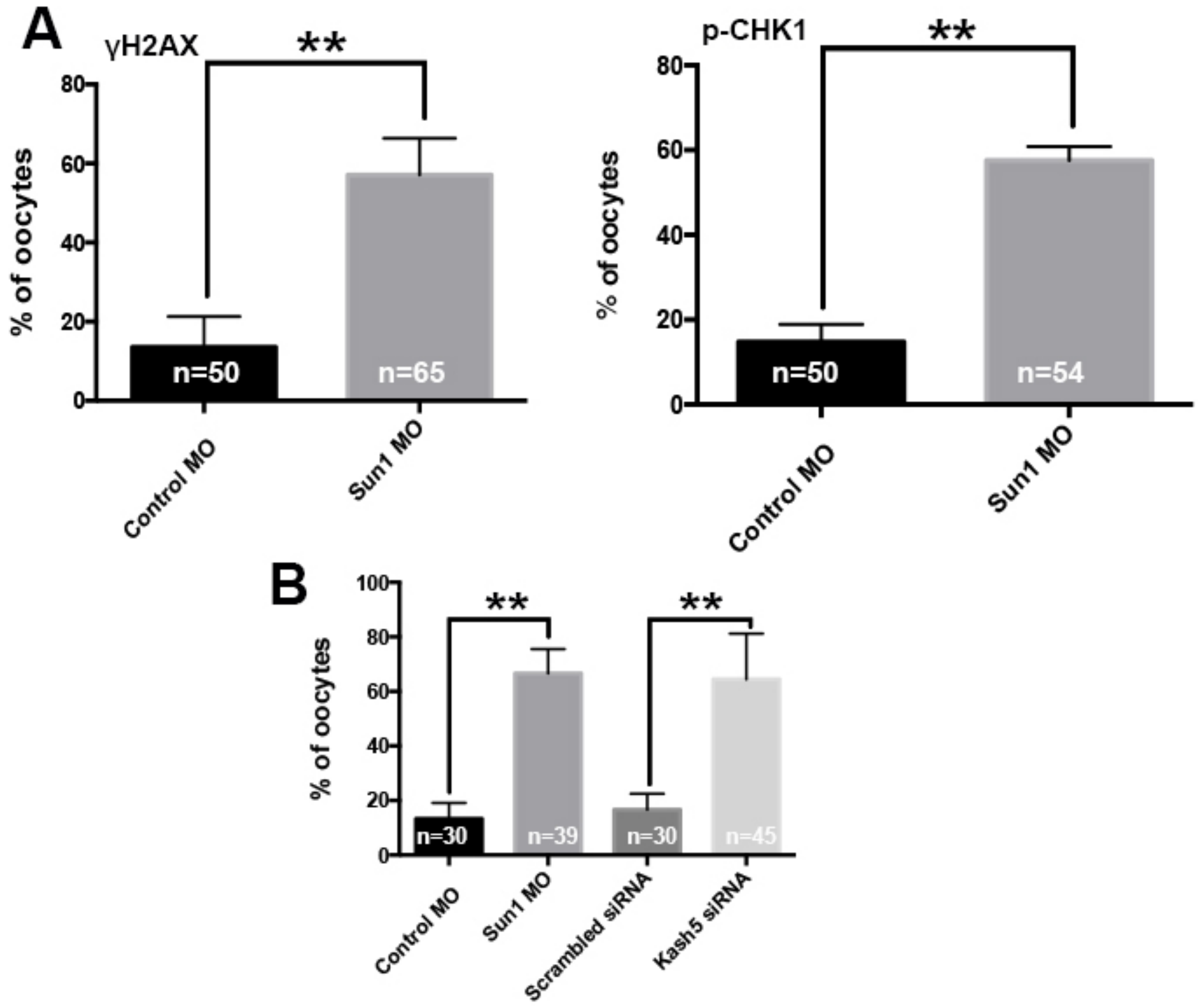

Figure S3. A, The statistical results of oocytes with positive γH2AX and p-CHK1 signals. B, The statistical results of oocytes with migrated spindle after cultured for 7h. ** means $p<0.001$.
